# Volumetric Quantitative Ablation Margins for Assessment of Ablation Completeness in Thermal Ablation of Liver Tumors

**DOI:** 10.3389/fonc.2021.623098

**Published:** 2021-03-10

**Authors:** Raluca-Maria Sandu, Iwan Paolucci, Simeon J. S. Ruiter, Raphael Sznitman, Koert P. de Jong, Jacob Freedman, Stefan Weber, Pascale Tinguely

**Affiliations:** ^1^ ARTORG Center for Biomedical Engineering Research, University of Bern, Bern, Switzerland; ^2^ Department of Hepato-Pancreato-Biliary Surgery and Liver Transplantation, University Medical Center Groningen, Groningen, Netherlands; ^3^ Division of Surgery, Department of Clinical Sciences, Karolinska Institutet at Danderyd Hospital, Stockholm, Sweden; ^4^ Department of Visceral Surgery and Medicine, Inselspital University Hospital Bern, University of Bern, Bern, Switzerland

**Keywords:** liver neoplasms, ablation techniques, computer-assisted therapies, stereotactic techniques, interventional radiology

## Abstract

**Background:**

In thermal ablation of liver tumors, complete coverage of the tumor volume by the ablation volume with a sufficient ablation margin is the most important factor for treatment success. Evaluation of ablation completeness is commonly performed by visual inspection in 2D and is prone to inter-reader variability. This work aimed to introduce a standardized approach for evaluation of ablation completeness after CT-guided thermal ablation of liver tumors, using volumetric quantitative ablation margins (QAM).

**Methods:**

A QAM computation metric based on volumetric segmentations of tumor and ablation areas and signed Euclidean surface distance maps was developed, including a novel algorithm to address QAM computation in subcapsular tumors. The code for QAM computation was verified in artificial examples of tumor and ablation spheres simulating varying scenarios of ablation margins. The applicability of the QAM metric was investigated in representative cases extracted from a prospective database of colorectal liver metastases (CRLM) treated with stereotactic microwave ablation (SMWA).

**Results:**

Applicability of the proposed QAM metric was confirmed in artificial and clinical example cases. Numerical and visual options of data presentation displaying substrata of QAM distributions were proposed. For subcapsular tumors, the underestimation of tumor coverage by the ablation volume when applying an unadjusted QAM method was confirmed, supporting the benefits of using the proposed algorithm for QAM computation in these cases. The computational code for developed QAM was made publicly available, encouraging the use of a standard and objective metric in reporting ablation completeness and margins.

**Conclusion:**

The proposed volumetric approach for QAM computation including a novel algorithm to address subcapsular liver tumors enables precision and reproducibility in the assessment of ablation margins. The quantitative feedback on ablation completeness opens possibilities for intra-operative decision making and for refined analyses on predictability and consistency of local tumor control after thermal ablation of liver tumors.

## Introduction

Image-guided thermal ablation using microwaves (MWA) or radiofrequency (RFA) is an established tissue-sparing, low-morbidity treatment techniques for malignant liver tumors (1). Frequently applied imaging modalities include ultrasound, computed tomography (CT), or magnetic resonance imaging (MRI), which allow high quality guidance in a minimally invasive treatment environment. To further increase targeting accuracy, safety, and treatment reproducibility, stereotactic navigation systems have been introduced and the results reported in clinical studies (2–5).

The most important factor defining treatment success in thermal ablation is a complete coverage of the targeted tumor by the ablation volume, with a sufficient ablation margin. The latter was confirmed to be an independent predictor of ablation site recurrence (ASR) in several studies (6–11). The recommended minimal ablation margin is currently defined as 5 mm, a 10 mm margin being preferred (1, 7, 9). At present, the ablation margin is most commonly assessed visually, either using side-by-side juxtaposition of pre- and post-ablation images or using image-fusion, and reported in 2D at the site of largest tumor diameter (3, 4, 6, 12). The disadvantages of such evaluation include an accuracy limited to one 2D image, which is prone to subjectivity and inter-reader variability, even when performed by experienced radiologists (13).

To address these issues, the evaluation of ablation margins in a quantitative, volumetric manner has been proposed, including a commercially available product (Ablation-fit™, R.A.W SRL, Milano, Italy), applying (semi)- automatic segmentation of tumor and ablation volumes and registration of pre- and post-interventional images (12, 14–18). However, several important challenges remain for application of these methods for assessment of quantitative ablation margins (QAM) on a broader scale. Firstly, the description of circumferential ablation margins for tumors in close vicinity to the liver capsule cannot be accomplished due to the inherently limited surrounding liver parenchyma. These cases are rarely addressed in the literature (12, 13), and no detailed technical solution for the computation of QAM for subcapsular tumors exists to date. Secondly, most studies focus on an average minimal ablation margin, whereas the assessment of a quantitative distribution of margin distances could take the accuracy of QAM one step further. Thirdly, the lack of available codes or software packages alongside the implementation descriptions currently limits reproducibility of the described approaches.

The aim of this work was to introduce a method for increased accuracy and standardized evaluation of ablation margins, for future intra-operative feedback on the ablation completeness during image-guided thermal ablation. To this end, a quantitative volumetric approach for assessment of ablation margins based on surface distance maps and an algorithm to address subcapsular tumors were developed. This work presents the computational pipeline and experimental evaluation of the QAM metric, using artificial examples and clinical data from selected patients treated with stereotactic microwave ablation (SMWA) for colorectal liver metastases (CRLM).

## Material and Methods

### Development of Quantitative Ablation Margin Metric

#### Requirements on Input Data

The requirements regarding input data to compute the QAM metric included i) pre- and post-ablation scans co-registered and resampled to the same space and size (image fusion). Ideally, this is achieved during image acquisition by means of patient fixation (*e.g.* vacuum mattress), reduction of breathing motion (*e.g.* jet ventilation or controlled apnea) and image parameter selection, factors which are typically achieved during stereotactic CT-guided ablation procedures (2), ii) tumor and ablation volumes segmented and saved as binary segmentation masks. For subcapsular tumors, segmentation of the liver capsule surrounding the liver tumor (at least 5 mm larger than the tumor), iii) availability of all segmented masks with the same dimensions in x, y, z directions and the same voxel spacing.

#### Computation Pipeline

**Figure 1:**
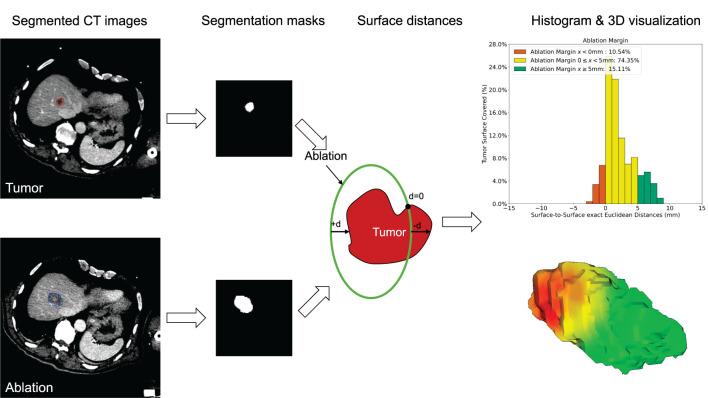
Quantitative ablation margin (QAM) computation pipeline.

Ablation margins are clinically defined as the area of necrosis extending from the tumor surface to the ablation surface. The proposed QAM method computes the distances from each tumor surface voxel to the closest ablation surface voxel. To this end, the exact Euclidean distance transform (19) was first applied to the surface voxels of the ablation segmentation mask. Distances were then extracted using the surface voxels of the tumor segmentation mask. This method was based on the surface Dice-Sørensen Coefficient, which measures the agreement between two surfaces (20). The algorithm was adapted by using a signed distance map to distinguish between positive and negative margins, and an “exclusion volume” to exclude tumor voxels near the liver surface in case of subcapsular tumors. The QAM computation pipeline is illustrated in **Figure 1**.

More specifically, the calculations encompass the following step-by-step algorithm:

1. Loading of input data (see **Figure 1**), consisting of
  1.1. Tumor Segmentation
  1.2. Ablation Segmentation
  1.3. Liver Segmentation (exclusion volume for subcapsular cases)
2. Computation of the maximum bounding box enclosing all input segmentations from Step 1 and cropping the volumes to the smallest possible processing sub-volume (optional step to decreases computation time without impacting the quality of the results)
3. Computation of the contours of the cropped tumor, ablation, and liver segmentations. Subtraction of a morphologically eroded mask, using a structuring element with a three-by-one shape to extract only face connected surface voxels (six-connected). This ensures the contour being as “thin” as possible (only one layer of voxels) and avoid exclusion of corners where large edges are encountered in the segmentation.
4. Computation of the exact signed surface-to-surface Euclidean distance on the ablation segmentation using the image spacing. The exact Euclidean distance transform function from SciPy (21) is employed to measure the closest distance from each voxel to a voxel of the ablation mask contour, by considering the voxel size of the image along each direction. The distance is given by
  $$y_{i}=\sqrt{\sum_{i}^{n}{\left(x_{i}-b_{i}\right)}^{2}},$$
  where *b_i_* is the background value, *x_i_* is the foreground input point and *n* is the number of dimensions.
5. Labeling of all background voxels (0/False) in the ablation segmentation mask with −1 to create a signed distance map. The latter is designed to define negative distances, *i.e.* tumor voxels that are outside the ablation volume. Subsequent voxel-wise multiplication of this ablation segmentation mask with the distance map obtained at the previous step.
6. For subcapsular tumors: computation of the exact Euclidean distance transform on the liver segmentation, setting all tumor surface voxels which are closer than 5 mm to the liver surface to 0 (see paragraph below).
7. Computation of the intersection between the tumor contour voxels and the signed ablation distance map to extract the tumor-to-ablation exact Euclidean surface distances.

#### The Special Case of Subcapsular Tumors

**Figure 2:**
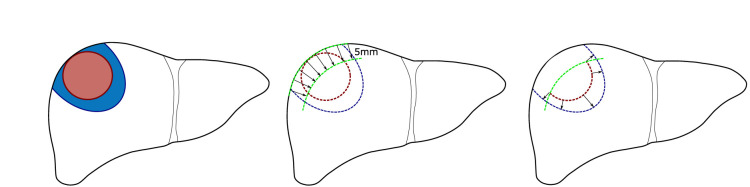
Schematic illustration of quantitative ablation margins (QAM) for subcapsular tumors. Left: Subcapsular tumor (red) surrounded by ablation volume (blue). Center: Exclusion volume of 5 mm below liver capsule. Right: Remaining tumor surface used for final QAM computation.

For subcapsular tumors, erroneously small ablation margins might arise in quantitative ablation measurements, due to the inherent impossibility of margins >5 mm if the tumor borders with the liver capsule. To this end, a method to account for potentially misleading negative or insufficient margins was developed. Next to the tumor and ablation volume, the local liver capsule surrounding the subcapsular tumor is segmented in these cases. This is performed with a minimum of 5 mm surrounding the tumor border, such that the tumor would not be discarded in the QAM calculation. Subsequently, all tumor voxels in less than 5 mm proximity to the liver capsule are excluded from the computation by setting their value to 0/False (background) (**Figure 2**). An exclusion volume threshold of 5 mm below the liver capsule was chosen following the most frequently accepted definition of minimal margin for complete ablation (6). However, this threshold could be modified according to individual treatment targets.

#### Data Display and Visualization

The QAM output data are displayed i) numerically as the maximum, minimum, and the 25^th^ percentile, 50^th^ percentile (median) and 75^th^ percentile of calculated surface distances, and ii) graphically as histograms displaying relative percentages of ablation margin distances, *i.e.* tumor surface coverage in substrata of 1 mm bins. The traffic light-colored histogram with orange, yellow, and green represent ablation margins of x < 0 mm, 0 ≥ x < 5 mm, and x ≥ 5mm, respectively. This range was selected based on the current literature (6) but could be modified according to clinical considerations. Additionally, surface distances are projected onto the ablation volume surface to visualize the areas of critical ablation margins in a 3D model, applying the same color scheme.

#### Code

NiBabel version 3.1.1 (https://nipy.org/nibabel/) was used to load the input data passed as Neuroimaging Informatics Technology Initiative (NiFTI) images to the main starting script, *qam/main.py*. The Region-of-Interest (ROI) images were further converted into arrays using NumPy version 1.19. To compute the surface distances, the script *qam/margin.py* was called as a function. Subsequently, the segmentation contours were extracted using the morphological operation erosion with kernel size generated by setting the structuring element as *ndimage.generate_binary_structure(3,1)* from SciPy version 1.5.1. The exact surface-to-surface Euclidean distances were computed with *scipy.ndimage.morphology.distance_transform_edt* as implemented in SciPy version 1.5.1. Histogram visualizations were displayed with Seaborn version 0.10.1 (https://seaborn.pydata.org/) and Matplotlib version 3.2.2. This code is located in the file *qam/plotting.py*. The CT images shown in the *Results* section were visualized using Mango version 4.1 (http://rii.uthscsa.edu/mango/). The 3D visualizations were created using VTK version 9.0.1 (www.vtk.org). This code is located in the file *qam/visualization.py*. An example for automated QAM computation over a larger dataset is located in the *examples* folder.

### QAM Validation Experiments

#### Artificial Cases

**Figure 3:**
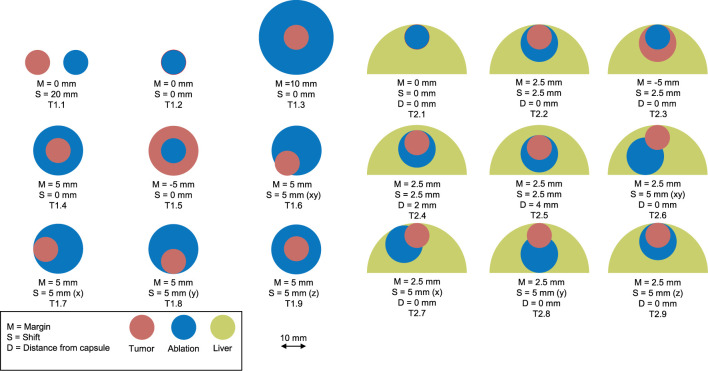
Artificial examples simulating tumor (red) and ablation (blue) volumes. Left: non-subcapsular tumors and ablation volumes. Right: subcapsular tumors with ablation volumes and their relationship to the liver capsule (green).

To evaluate the accuracy and precision of the developed QAM metric, a set of artificial input data was designed such that the output data could be validated by comparisons with manual calculation. These examples consist of spheres emulating tumors, ablations, and liver capsules (**Figure 3**). All examples were created programmatically and stored in the data format described in section ‘*Requirements on Input Data’*. Two representative cases were selected to illustrate QAM. Case T1.6 represents a 10 mm tumor which was ablated with a 15 mm ablation zone shifted by 5 mm in x and y directions, leaving an area of tumor with a negative margin. Case T2.2 represents a subcapsular tumor with diameter of 10 mm and a 15 mm ablation zone covering the entire tumor. This case was used to assess the difference in QAM distribution with and without the exclusion volume for subcapsular tumors.

#### Clinical Cases

To investigate applicability of the proposed QAM metric on clinical ablation cases, a subset of representative patients treated with stereotactic microwave ablation (SMWA) was selected from a prospective database of a European multicenter cohort trial (MAVERRIC, Microwave Ablation Versus Resection for Resectable Colorectal Liver Metastases; clinicaltrials.gov: NCT02642185) (22). In this trial, all tumors were treated using stereotactic image-guidance systems (CAS-ONE IR, CAScination AG, Bern, Switzerland and NPS, DEMCON Advanced Mechatronics, Enschede, The Netherlands). Detailed set-up and procedural workflows of both systems have previously been described (2, 3, 23). To ensure minimal patient and liver movement, all interventions were performed under general anesthesia, using High Frequency Jet Ventilation (24) or controlled apnoea and optionally with patients positioned on a vacuum mattress. CT scans for qualitative ablation validation were acquired immediately after the ablation treatment and with the ablation probe withdrawn. Due to patient fixation and control of breathing motion, the tumor (planning scan) and ablation (ablation validation scan) CT scans were co-registered (image-fusion). For segmentation of tumor and ablation volumes, and the surrounding liver capsule in case of subcapsular tumors (defined as a distance to the liver capsule of ≤5 mm), a semi-automatic segmentation tool was used in Amira (Amira 6.3, ThermoFisher Scientific, USA). Segmentation volumes were verified by experienced radiologists from the respective clinical institutions. Images from each tumor dataset were converted into the input format described in *Requirements on Input Data* section, resampled to the same size and voxel spacing as the final validation CT scan and saved as NiFTI image format.

## Results

### Code

The code for QAM is published as an open-source Python (version 3.8.2) repository under https://github.com/artorg-unibe-ch/qam. The code can be either integrated into existing software packages or used as a stand-alone command line tool. Additionally, the repository contains the code to generate the artificial examples presented in the experimental section, in the file “create_qam_test_cases.py”.

### Artificial Cases

**Figure 4:**
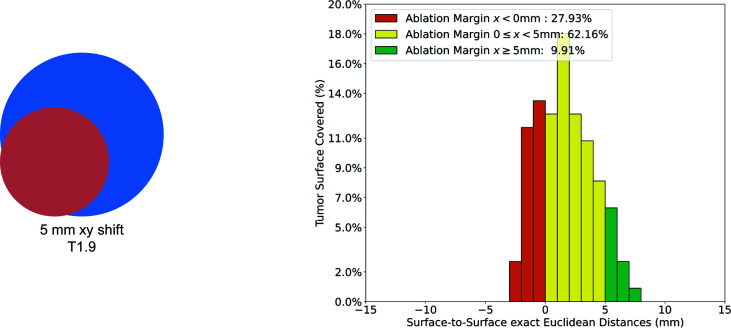
Visualization of QAM histogram for non-subcapsular artificial case T1.6. Left: artificial example with tumor (red) shifted 5 mm in the x and y directions with respect to the ablation (blue). Right: Histogram of relative distribution of margin distances displayed as 1 mm substrata.

To assess the feasibility of the QAM metric, the QAM code was run for all artificial examples and validated against manual computations. The numerical results of all artificial cases can be found in the supplementary material and the public code repository. The results for the artificial case T1.6 are described numerically and visually displayed below (**Table 1**, **Figure 4**). In this example, the ablation sphere was shifted by 5 mm in both x and y directions, which lead to 27.93% of the tumor surface not being covered (QAM x < 0 mm), 62.16% of the tumor surface being covered with QAM 0 ≤ x < 5 mm and 9.91% of the tumor surface being covered with a margin ≥ 5 mm.

**Table 1:** The quantitative ablation margin (QAM) results for non-subcapsular case T1.6.

Case ID		T1.6	
Euclidean distance (mm)		Percentage of tumor surface covered at distance	
Min	−2.23	x < 0	27.93%
25^th^ percentile	−1	x ≥ 0 < 5	62.16%
Median	1	x ≥ 5	9.91%
75^th^ percentile	3		
Max	7		

**Figure 5:**
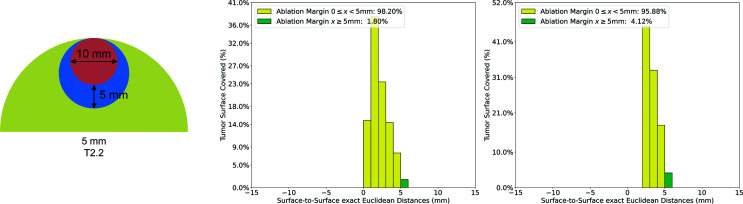
Quantitative ablation margin (QAM) computation for subcapsular artificial case T2.2. with and without applying the algorithm for subcapsular tumors. Left: T2.2 artificial example (tumor: red, ablation: blue, liver: green). Center: Relative distribution of margins with exclusion volume not subtracted in QAM computation. Right: Relative distribution of margins with exclusion volume subtracted in QAM computation.

To demonstrate how the algorithm for subcapsular tumors influenced QAM distribution, the QAM results for case T2.2 with and without subtraction of a 5mm exclusion volume are shown in **Figure 5**. The QAM 0 ≤ x < 5 mm changed from 98.2% to 95.88%, and the QAM ≥ 5 mm from 1.80% to 4.12%, when a 5 mm subcapsular volume was subtracted.

### Clinical Cases

The QAM code was run successfully for all clinical cases extracted for margin analysis from the available study cohort (n tumors = 65). Four representative cases were selected to assess the applicability of using QAM for volumetric assessment of ablation completeness in CRLM cases treated with SMWA.

**Figure 6:**
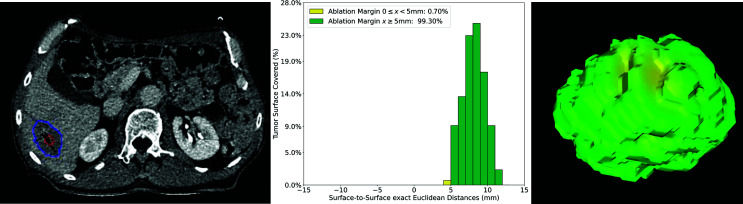
Volumetric assessment of quantitative ablation margins (QAM) in a patient treated with SMWA for a CRLM in liver segment VI. Left: Computed tomogram image with segmented tumor (red) and ablation (blue) volumes. Center: Relative frequencies of ablation margin substrata with respect to percentage of tumor surface covered. Right: 3D representation of the ablation margin projected onto the ablation surface.

While not explicitly measured, manual segmentation of tumor and ablation volumes as well as the liver capsule, if required, varied between 5 and 30 min depending on the quality of contrast-enhancement of the CT scans. Using the provided code, the time for QAM computation including visualizations, depending on the lesion size, was generally performed in under 30 s using a standard notebook. **Figure 6** illustrates the volumetric QAM assessment of a patient treated with SMWA for a CRLM lesion located in liver segment VI. In this case, SMWA resulted in QAM ≥5 mm in 99.3% and QAM 0 ≥ x < 5 mm in 0.7%. The majority of tumor surface (24%) was covered by an ablation margin of 8mm, with decreasing frequencies of QAM > 8 mm to a maximum of 11 mm (3%), and of QAM <8 mm to a minimum of 4 mm (1%).

**Figure 7:**
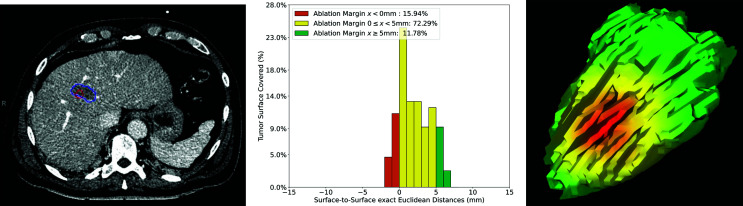
Volumetric assessment of quantitative ablation margins (QAM) in a patient treated with SMWA for a CRLM in liver segment VIII. Left: Computed tomogram image with segmented tumor (red) and ablation (blue) volumes. Center: Relative frequencies of ablation margin substrata with respect to percentage of tumor surface covered. Right: 3D representation of the ablation margin projected onto the ablation surface, showing the area of negative margins in red.

**Figure 7** shows a case with a patient treated with SMWA for a CRLM in segment VIII, located adjacent to a segment VIII portal vein branch. In this example, the QAM metric resulted in a tumor surface coverage rate ≥5 mm of 11.8% and 0 ≤ x < 5 mm of 72.3%. A total tumor surface of 15.9% was ablated with negative margins (QAM < 0 mm), of which 11.3% with −1 mm and 4.6% with −2 mm. As visible from the CT image, the ablation volume was of irregular, non-ellipsoid shape, with QAM ranging from −1.8 mm to 6.8 mm, the main relative frequency of QAM being 0 mm (28%). **Table 2** shows a numerical presentation of this clinical example, including minimal, maximal, and interquartile range values.

**Table 2:** Numerical representation of the ablation margin of case presented in **Figure 7**.

Case ID		**Figure 7**	
Euclidean distance (mm)		Percentage of tumor surface covered at distance	
Min	−1.8	x < 0	15.94%
25^th^ percentile	0	x ≥ 0 < 5	72.29%
Median	1.8	x ≥ 5	11.78%
75^th^ percentile	3.9
Max	6.8

**Figure 8:**
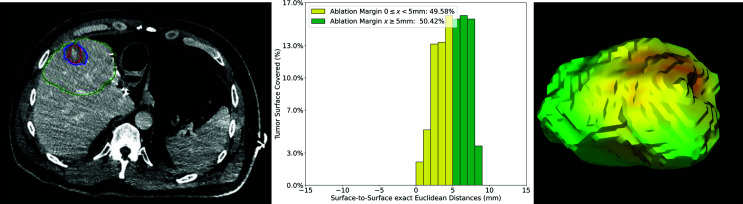
Volumetric assessment of quantitative ablation margins (QAM) of a subcapsular CRLM in liver segment V. Left: Computed tomogram with segmented tumor (red), ablation (blue) volumes and the local liver capsule surrounding the tumor (green). Center: Relative frequencies of ablation margin substrata with respect to percentage of tumor surface covered. Right: 3D representation of the ablation margin projected onto the ablation surface.

The volumetric QAM assessment after SMWA for a CRLM tumor located in a subcapsular position in segment V is illustrated in **Figure 8**. In this example, a median QAM of 5.05 mm was reached, with an interquartile range from 3.35 to 6.56 mm. A QAM of 0 ≤ x < 5 mm 49.6% and QAM ≥ 5 mm of 50.4% were reached. Next to the distribution of relative QAM frequencies, a visualization of the exact location of the QAM levels in 3D space is shown and displayed in the respective traffic-light colors.

**Figure 9:**
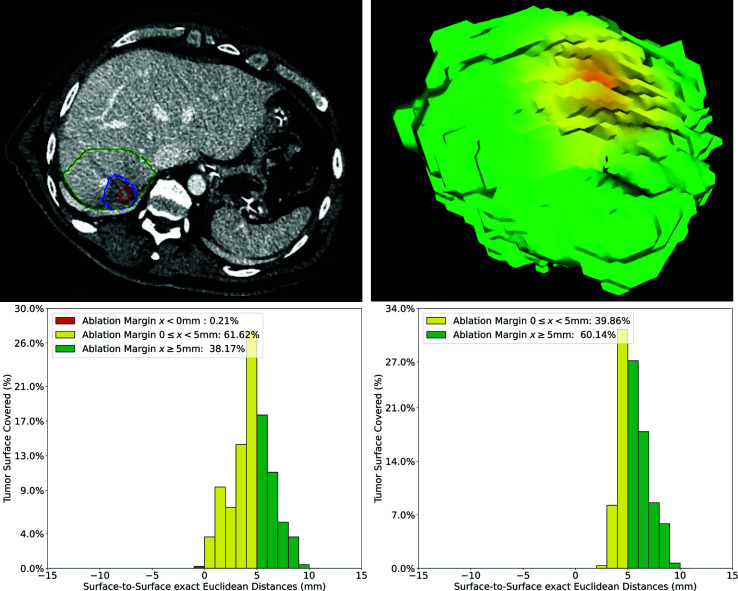
Volumetric assessment of quantitative ablation margins (QAM) of a subcapsular CRLM in liver segment VII. Upper left: Computed tomogram with segmented tumor (red), ablation (blue) volumes and the local liver capsule surrounding the tumor (green). Upper right: 3D representation of the ablation margin projected onto the ablation surface. Lower left: Histogram of ablation margin substrata without applying algorithm for subcapsular tumors. Lower right: Relative frequencies of ablation margin substrata with respect to percentage of tumor surface covered when applying algorithm for subcapsular tumors.

In **Figure 9**, an example case of a CRLM located in a subcapsular position in liver segment VII is shown. To adjust for the subcapsular lesion location, the surrounding liver capsule was segmented and the computation of QAM adjusted as described. This tumor scored a QAM ≥ 5 mm of 39.7% and QAM 0 ≤ x < 5 mm of 60.1%. To display the impact of the proposed algorithm of QAM computation for subcapsular tumors, the QAM histogram without adaption for subcapsular tumors is additionally displayed. After applying the described QAM computation for subcapsular tumors, the minimum QAM changed from −1 to 3 mm, and the relative frequencies of QAM substrata were re-distributed accordingly.

## Discussion

This work proposes a method for quantifiable volumetric assessment of ablation completeness after thermal ablation of liver tumors. A computational pathway of quantitative ablation margins (QAM) was developed using a signed Euclidean surface distance map, including a novel algorithm to address subcapsular tumors. The computational code for applying the developed QAM metric was made publicly available, encouraging the use of a standardized approach and definition in reporting ablation margin coverage. Applicability of the proposed method was shown in artificial and clinical examples, presenting a numerical and visual way of displaying the QAM output.

The most important factor for treatment success after thermal ablation of liver tumors is a complete tumor coverage by the ablation volume (6, 12, 25). While nomenclature, time point, and metric of the assessment of ablation completeness vary in the current literature (26, 27), the necessity of an additional minimal ablation margin encompassing the targeted tumor is generally agreed upon (7, 9). The convention of defining a minimal treatment margin stems from surgical resection, where the histopathology result of the surgical specimen confidently quantifies the surgical safety margin, allowing refined analyses of subgroups of resection margins on tumor recurrence (28). Contrarily, in image-guided thermal ablation the assessment of treatment completeness is mainly performed by visual inspection of follow-up imaging. Even though a correspondence between radiologic and histopathologic findings in explanted livers was reported after radiofrequency ablation of hepatocellular carcinoma (29), a visual assessment without available histopathology results remains subjective and prone to inter-personal variations (13).

Toward a quantifiable result of treatment completeness after thermal ablation—similar to a resection margin after surgical resection—previous works have presented various approaches for quantitative assessment of ablation margins, using either surface-to-surface distances or volume overlaps. Hocquelet et al. (16) previously described the ablation surface margin as the distance between six connected background voxels to the foreground voxels. Kaye et al. (12) presented a volumetric approach by reporting the volume of insufficient tumor coverage. Tani et al. (18) defined the concept of super-imposing a Euclidean distance map over the 3D surface model of the ablation similar to our methodology. A commercially available software package (Ablation-fit™, R.A.W SRL, Milano, Italy) for intra-procedural volumetric ablation assessment is currently being investigated in clinical trials (15). As opposed to these works, the strength of the herein presented QAM metric is the public provision of the full code including a detailed description of QAM substrata distributions, as well as the introduction of an algorithm to address subcapsular tumors. Although few attempts have been made to address this challenge (12, 15), no systematic approach allowing robust replication has been described so far. We confirmed that ablation margins can be underestimated when applying the unadjusted QAM method in subcapsular tumors (**Figure 9**), with the proposed algorithm potentially leading to a more accurate measurement of the actual ablation completeness according to a set definition. The QAM method presented in this work was designed to be as generalizable as possible, by avoiding dependencies to a specific software platform for registration and segmentation, and by being applicable to any thermal ablation treatment (MWA, RFA) and CT-guided technique (conventional free-hand, stereotactic navigation). However, since the accurate registration of pre- and post-ablation imaging (image fusion) is a pre-requisite of the technique, such software tools would ideally be integrated into stereotactic image-guidance systems, where they could provide immediate quantitative feedback on ablation completeness and treatment success in a seamless workflow. The use of (semi-) automatic segmentation algorithms would need to be optimized and directly integrated into the navigation system’s algorithm, potentially taking intra-procedural knowledge of treatment completeness one step further. Similar to frozen sections during surgery, this could allow immediate re-treatment based on objective and quantitative feedback without time delay, and therefore enhance treatment efficacy rates.

At its current stage, the application of this technique requires not only an experienced radiologist but a technically oriented member to pre-process (*i.e.* register, segment and store) the data and run the computations. While this is applicable in highly specialized centers or within clinical trials, only an integration of these tools into software workflows will bring this technique to widespread clinical use. Outside of navigation systems, the technology would need to be integrated into dedicated radiology software, or provided as a standalone software package together with registration and segmentation tools. This would facilitate the conduction of clinical trials investigating optimal margin thresholds for different tumor types and potentially ablation procedures in other organs (*e.g.* lung, kidney).

Using QAM for accurate and reproducible assessment of ablation completeness leads to several further important aspects of local tumor control after thermal ablation of liver tumors. Firstly, the QAM output will allow to objectively differentiate between incomplete ablation and true ASR. In light of the subjectivity of conventional visual inspection of ablation completeness, ASR is currently defined as “the appearance of tumor foci at the edge of the ablation zone after at least one contrast-enhanced follow-up study has documented adequate ablation and an absence of viable tissue in the target tumor and surrounding ablation margin by using imaging criteria” (27). QAM will not only allow avoiding time delays in assessing ablation completeness, but enable the investigation of factors influencing true ASR as opposed to remaining incomplete ablation, arising on any follow-up imaging after initial ablation. This would greatly help to standardize the reporting of outcomes after thermal ablation of liver tumors. Secondly, QAM will allow in-depth analyses of correlation between ablation margins and ASR, toward the development of precision tools for prediction of local tumor control. It will allow refined analyses using distributions of margin substrata as opposed to a simple “minimal ablation margin”, a correlation of QAM with regard to ASR in 3D space, as well as interactions between QAM and other factors known to affect ASR such as tumor diameter or KRAS mutational status (25, 30). Without doubt, prospective trials will be required to evaluate the predictive value of the QAM metric with respect to ASR. Thirdly, QAM might serve as an objective endpoint to study factors associated with the expansion of ablation volumes, which remains to be poorly understood and highly dependent on individual tumor- and patient-related parameters.

A potential limitation of the proposed QAM algorithm to assess ablation completeness in subcapsular tumors is a limited applicability for very small tumors, *e.g.* <5 mm diameter, located within 5 mm of the liver capsule. In this case, all tumor voxels will be subtracted from the analysis, and no surface distances yielded. If the QAM metric would be adapted regarding the extent of subcapsular volume subtracted, a limited applicability for the respective size of subcapsular tumors would need to be kept in mind. Accordingly, a potential correlation between QAM computation for subcapsular tumors and local tumor progression would yield erroneous results if a recurrence occurred in the subcapsular area, which would need to be investigated in detail. While an alternative approach would have been to consider the tumor surface proximal to the liver capsule as completely ablated, we deliberately chose to subtract this area as to not potentially report overestimated ablation completeness rates. Furthermore, ablation of subcapsular lesions typically produces wedge-shaped ablation volumes, supporting the subcapsular exclusion threshold of 5 mm and a minimal lateral ablation margin as a criterion of success in these cases. A further application of such an exclusion volume from QAM computation would be for tumors adjacent to major blood vessels, where insufficient ablation margins might be reported due to the heat sink effect. Another potential limitation arises due to the fact that thermal ablation with MWA is known to cause significant tissue shrinkage from desiccation and protein denaturation immediately post treatment, with expansion of ablation volumes thereafter (15, 31, 32). This might lead to an underestimation of tumor coverage as assessed by QAM if computed on the immediate post-interventional scan. Also, even though the QAM metric was designed for use in all approaches of percutaneous ablation, it might be more difficult to apply if ablation is performed without stereotactic navigation, in which case software for deformable registration would be required (33). Since registration inaccuracies would lead to imprecise estimation of QAM, verifying registration accuracy before applying the QAM method is crucial. This said, image fusion prior to QAM computation would ideally be evaluated with an equally quantitative method to ensure accurate registration of pre- and post-ablation images, regardless of the type of targeting approach. It is of no doubt that the introduction of novel technologies such as SMWA and QAM bring additional efforts in the set-up and interventional workflows, the main challenge representing an optimal training of staff, after which learning curves are relatively fast and additional efforts can be reduced (34). First comparative studies further suggest enhanced treatment efficacy when using navigated ablation as opposed to conventional targeting (35), hence, overall treatment costs might be compensated if frequent re-treatments can be avoided. In conclusion, this work presents and publicly displays an algorithm for volumetric quantitative margin computation to assess ablation completeness after thermal ablation of liver tumors, including a novel algorithm to address subcapsular tumors. The objectified feedback on ablation completeness opens possibilities for intra-operative decision making and refined analyses on predictability and consistency of local tumor control after thermal ablation of liver tumors.

## Data Availability Statement

The data analyzed in this study is subject to the following licenses/restrictions: artificial examples are available in the code repository (https://github.com/artorg-unibe-ch/qam). The data from the clinical examples are available upon request. Requests to access these data sets should be directed to the corresponding authors.

## Ethics Statement

Written informed consent was obtained from the individual(s) for the publication of any potentially identifiable images or data included in this article.

## Author Contributions

R-MS and IP: algorithm development, data collection, analysis and interpretation, and manuscript writing. SR: data collection, analysis and interpretation, and manuscript proofreading. RS, JF, and SW: study planning, analysis and interpretation, and manuscript proofreading. PT: data collection, analysis and interpretation, and manuscript writing. All authors contributed to the article and approved the submitted version.

## Funding

This project was partially funded by the H2020-MSCA-ITN Project No. 722068 HiPerNav (R-MS, SW, RS, and PT), by the Swiss Cancer League (PT) Grant Nr: BIL KLS-4894-08-2019, and by the Professor Dr. Max Cloëtta Foundation (PT). All funding institutions had no contribution in the design and execution of this project.

## Conflict of Interest

SW is co-founder and shareholder of CAScination, the manufacturer of one of the navigation systems applied for stereotactic microwave ablation of colorectal liver metastases in the clinical example cases analyzed in this study.

The remaining authors declare that the research was conducted in the absence of any commercial or financial relationships that could be construed as a potential conflict of interest.
