# Supplementary material for: Volumetric Quantitative Ablation Margins for Assessment of Ablation Completeness in Thermal Ablation of Liver Tumors
Source: Front Oncol. 2021 Mar 10;11:623098. doi: 10.3389/fonc.2021.623098 (PMC7988092; doi:10.3389/fonc.2021.623098)
Supplement: Supplementary file 1 [file DataSheet_1.pdf]

Supplementary Material

Artificial examples and results for non-subcapsular tumors

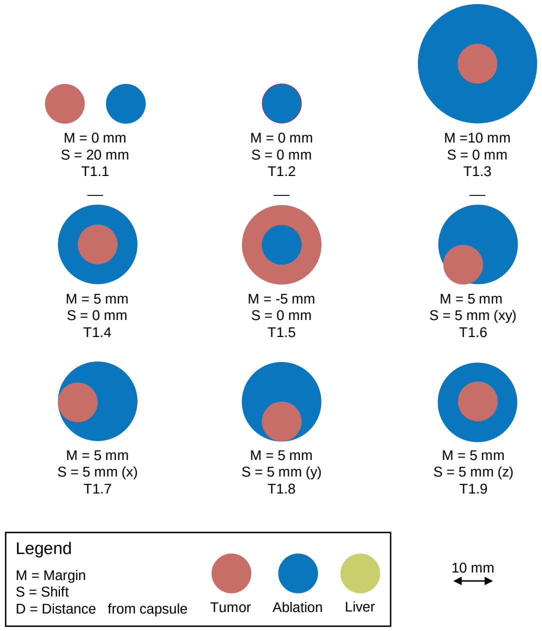

Figure 1: Artificial examples of non-subcapsular tumors

Numerical Results

Case	Euclidean distance (mm)					Percentage of tumor surface covered at x surface distances (mm)		
	Min	25th percentile	Median	75th percentile	Max	x < 0	x ≥ 0 < 5	x ≥5
T1.1	-20.00	-17.58	-15.65	-13.64	-10.00	100.00%	0.00%	0.00%
T1.2	0.00	0.00	0.00	0.00	0.00	0.00%	100.00%	0.00%
T1.3	9.06	9.64	9.70	9.95	10.00	0.00%	0.00%	100.00%
T1.4	4.12	4.58	4.69	5.00	5.00	0.00%	67.57%	32.43%
T1.5	-5.20	-5.10	-4.90	-4.58	-4.12	100.00%	0.00%	0.00%
T1.6	-2.24	-1.00	1.00	3.00	7.00	27.93%	62.16%	9.91%
T1.7	0.00	1.00	2.83	4.36	9.06	0.00%	79.73%	20.27%
T1.8	0.00	1.00	2.83	4.36	9.06	0.00%	79.73%	20.27%
T1.9	0.00	1.00	2.83	4.36	9.06	0.00%	79.73%	20.27%

Graphical results

T1.1

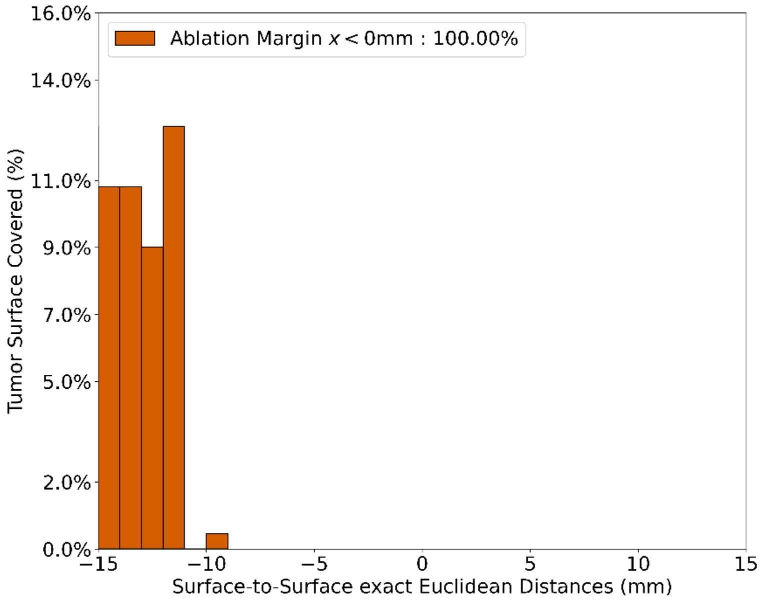

T1.2

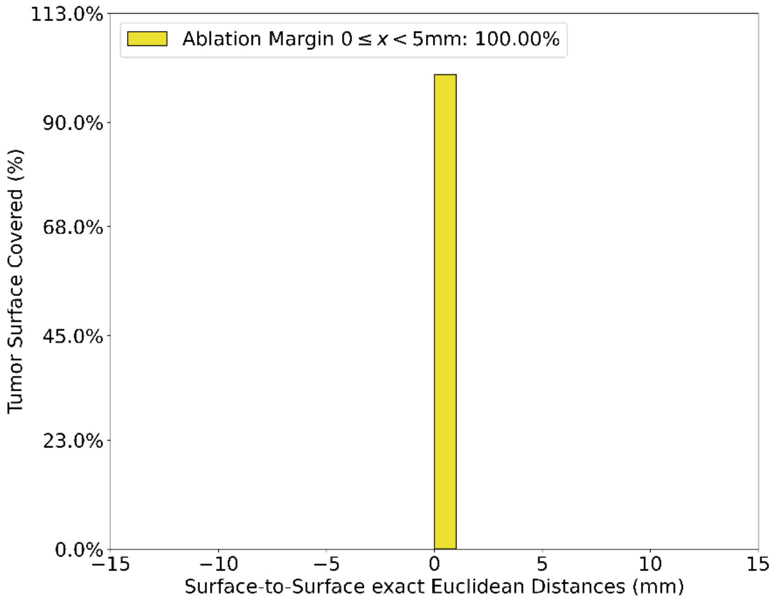

T1.3

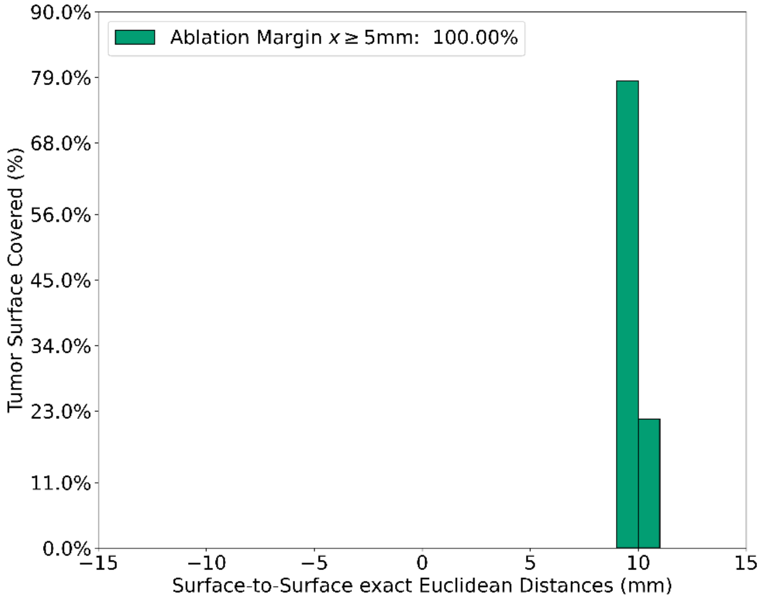

Ablation Margin $x \geq 5$mm: 100.00%
90.0%
79.0%
68.0%
56.0%
45.0%
34.0%
23.0%
11.0%
0.0%
Tumor Surface Covered (%)
−15
−10
−5
0
5
10
15
Surface-to-Surface exact Euclidean Distances (mm)

T1.4

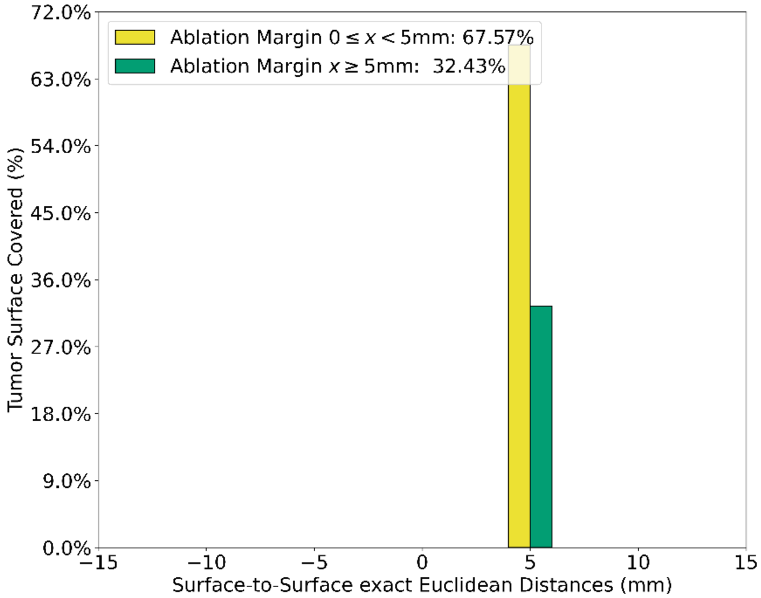

Ablation Margin $0 \leq x < 5$mm: 67.57%
Ablation Margin $x \geq 5$mm: 32.43%
72.0%
63.0%
54.0%
45.0%
36.0%
27.0%
18.0%
9.0%
0.0%
Tumor Surface Covered (%)
−15
−10
−5
0
5
10
15
Surface-to-Surface exact Euclidean Distances (mm)

T1.5

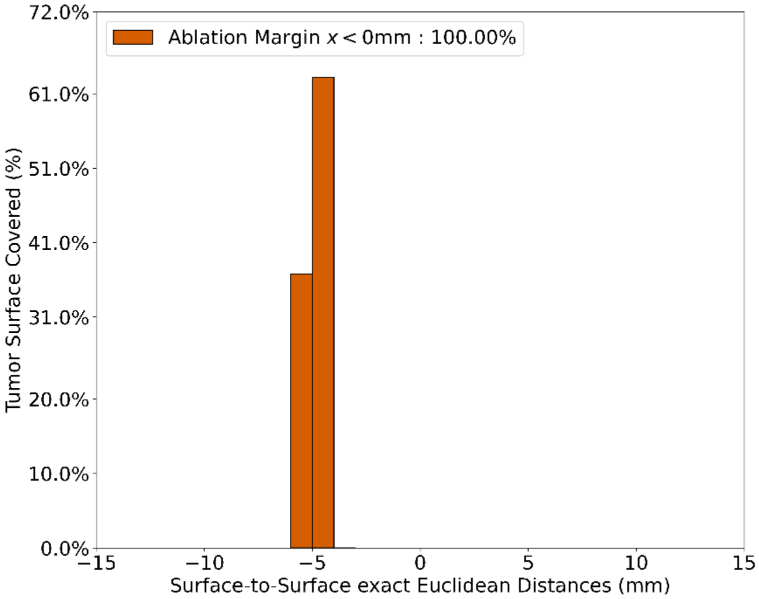

Ablation Margin $x < 0$mm : 100.00%
72.0%
61.0%
51.0%
41.0%
31.0%
20.0%
10.0%
0.0%
Tumor Surface Covered (%)
−15
−10
−5
0
5
10
15
Surface-to-Surface exact Euclidean Distances (mm)

T1.6

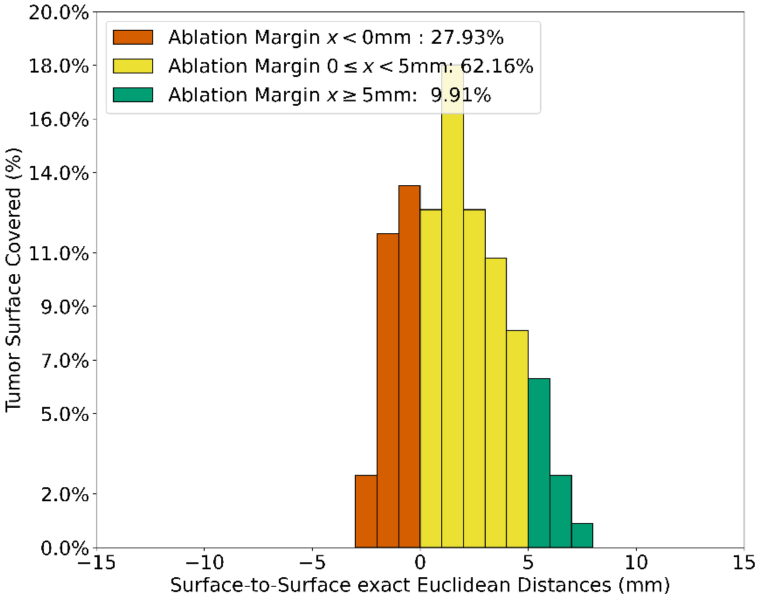

Ablation Margin $x < 0$mm : 27.93%
Ablation Margin $0 \leq x < 5$mm: 62.16%
Ablation Margin $x \geq 5$mm: 9.91%
20.0%
18.0%
16.0%
14.0%
11.0%
9.0%
7.0%
5.0%
2.0%
0.0%
Tumor Surface Covered (%)
−15
−10
−5
0
5
10
15
Surface-to-Surface exact Euclidean Distances (mm)

T1.7

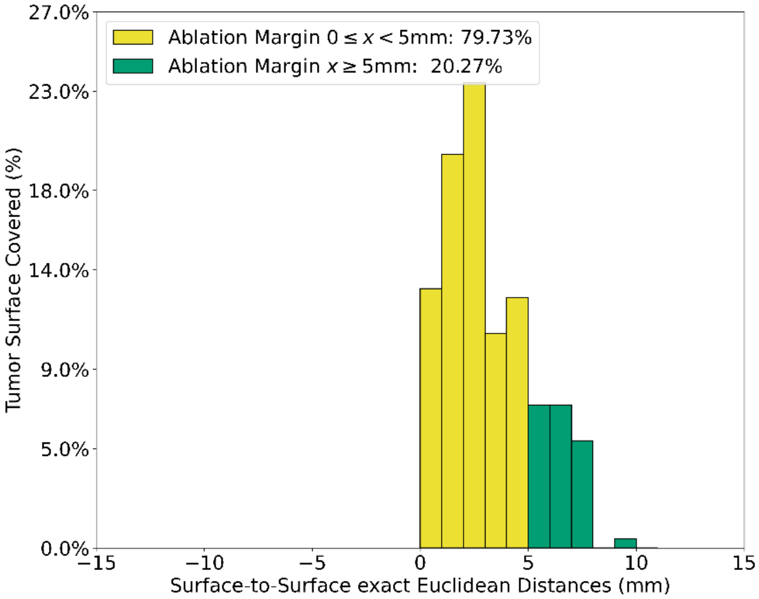
Ablation Margin 0 ≤ x < 5mm: 79.73%
Ablation Margin x ≥ 5mm: 20.27%
27.0%
23.0%
18.0%
14.0%
9.0%
5.0%
0.0%
Tumor Surface Covered (%)
−15
−10
−5
0
5
10
15
Surface-to-Surface exact Euclidean Distances (mm)

T1.8

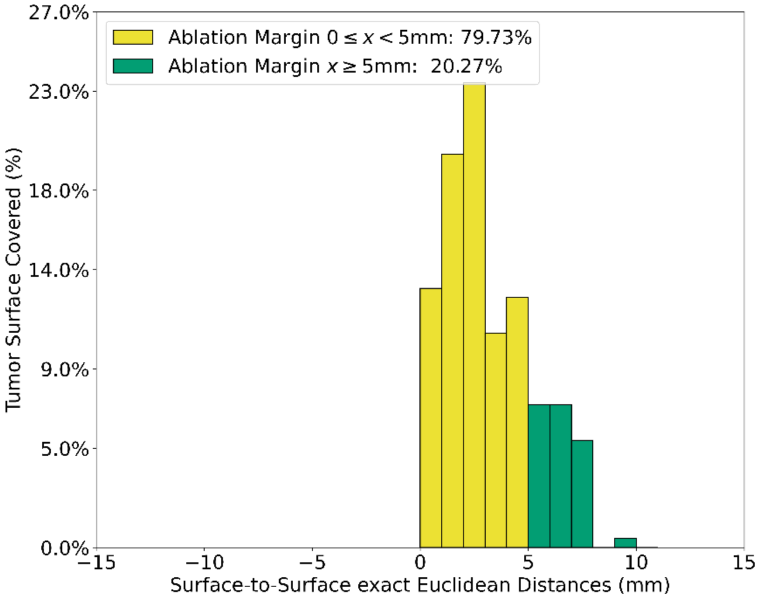
Ablation Margin 0 ≤ x < 5mm: 79.73%
Ablation Margin x ≥ 5mm: 20.27%
27.0%
23.0%
18.0%
14.0%
9.0%
5.0%
0.0%
Tumor Surface Covered (%)
−15
−10
−5
0
5
10
15
Surface-to-Surface exact Euclidean Distances (mm)

T1.9

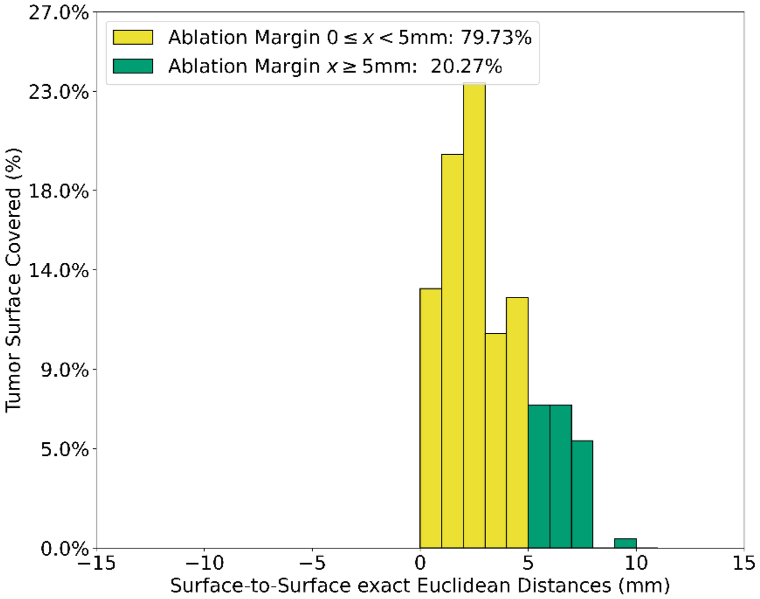

Ablation Margin 0 ≤ x < 5mm: 79.73%
Ablation Margin x ≥ 5mm: 20.27%
27.0%
23.0%
18.0%
14.0%
9.0%
5.0%
0.0%
Tumor Surface Covered (%)
−15
−10
−5
0
5
10
15
Surface-to-Surface exact Euclidean Distances (mm)

Artificial examples and results for subcapsular tumors

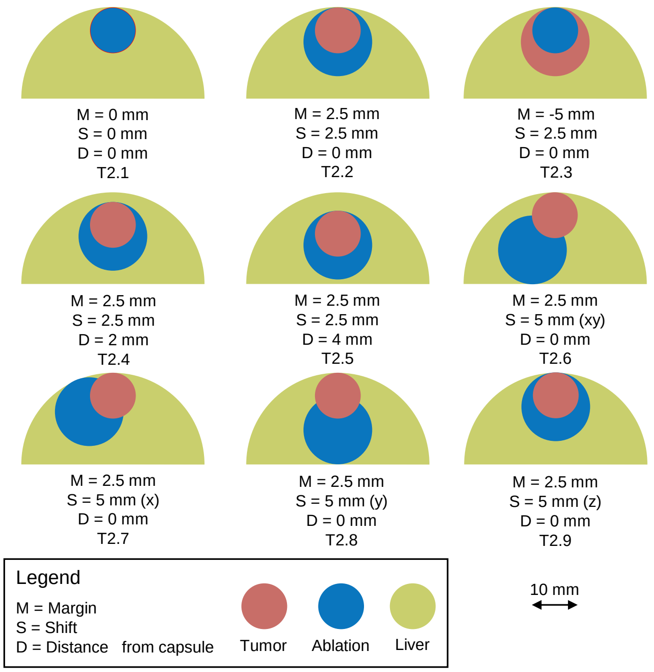

Figure 2: Artificial examples of subcapsular tumors

Table 2. Quantitative ablation margins (QAM) for results for synthetic subcapsular tumors

Case	Euclidean distance (mm)					% of tumor surface covered at x surface distances (mm)		
	Min	25th percentile	Median	75th percentile	Max	x < 0	x ≥ 0 < 5	x ≥5
T2.1	0.00	0.00	0.00	0.00	0.00	0.00%	100.00%	0.00%
T2.2	2.24	2.45	3.00	3.74	5.00	0.00%	95.88%	4.12%
T2.3	-5.10	-4.24	-3.74	-3.16	-2.24	100.00%	0.00%	0.00%
T2.4	1.41	1.73	2.45	3.16	5.00	0.00%	97.32%	2.68%
T2.5	0.00	1.41	2.24	3.00	5.00	0.00%	97.93%	2.07%
T2.6	-3.00	-1.00	1.00	2.45	4.47	32.99%	67.01%	0.00%
T2.7	-1.00	0.00	1.00	2.45	4.12	24.74%	75.26%	0.00%
T2.8	-2.00	-1.00	1.00	3.00	5.74	26.80%	68.05%	5.15%
T2.9	-2.00	-1.00	1.00	3.00	5.74	26.80%	68.05%	5.15%

Results

T2.1

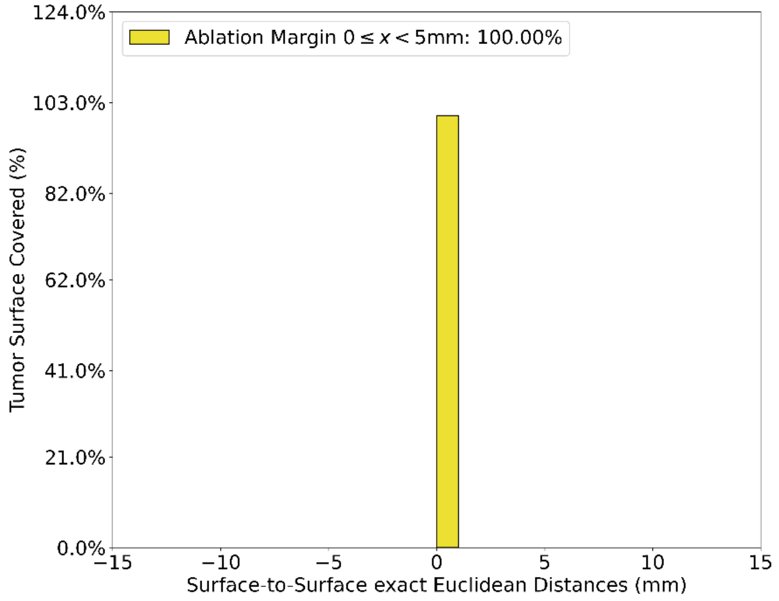

T2.2

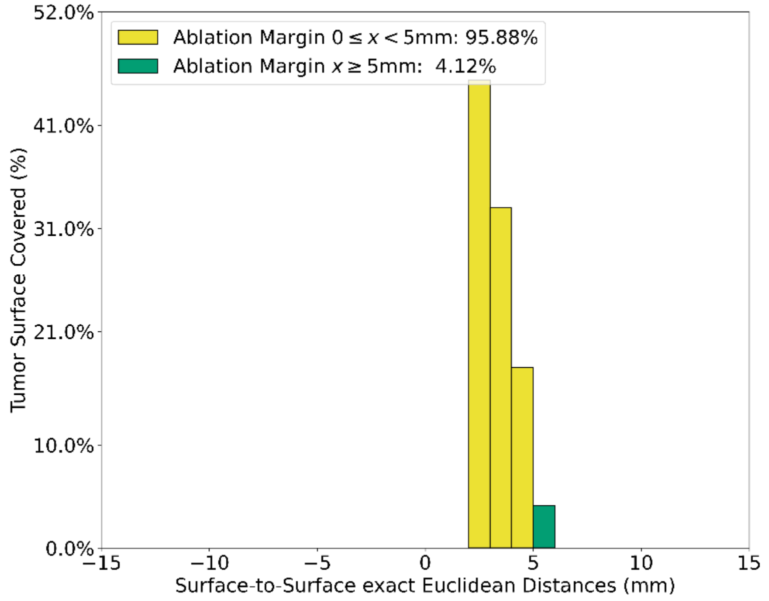

T2.3

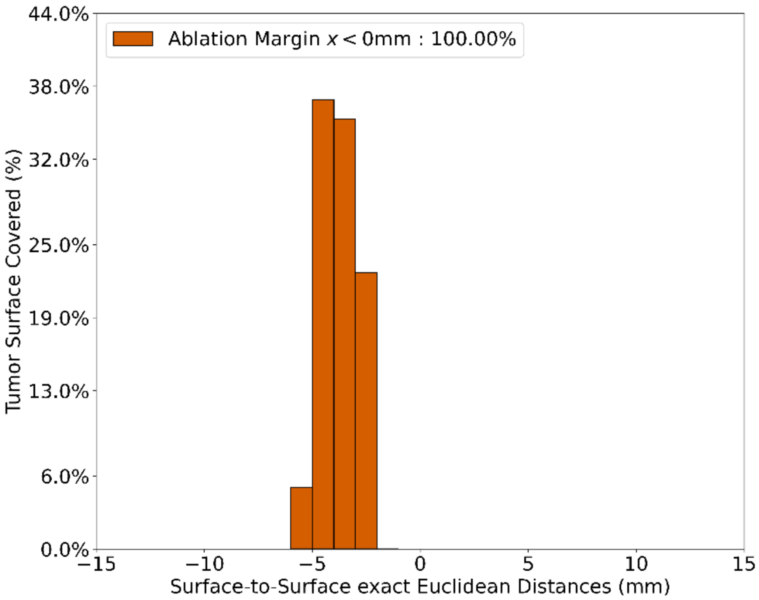
Ablation Margin $x < 0$mm : 100.00%
44.0%
38.0%
32.0%
25.0%
19.0%
13.0%
6.0%
0.0%
Tumor Surface Covered (%)
−15
−10
−5
0
5
10
15
Surface-to-Surface exact Euclidean Distances (mm)

T2.4

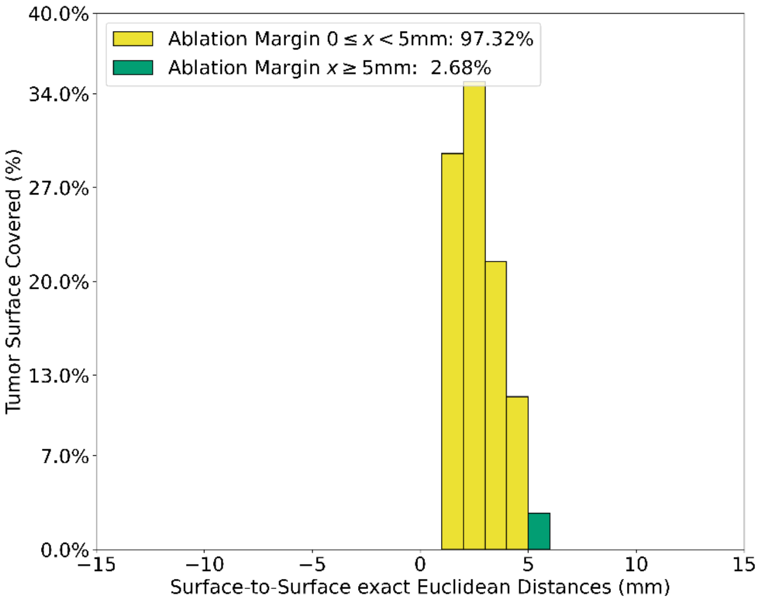
Ablation Margin $0 \leq x < 5$mm: 97.32%
Ablation Margin $x \geq 5$mm: 2.68%
40.0%
34.0%
27.0%
20.0%
13.0%
7.0%
0.0%
Tumor Surface Covered (%)
−15
−10
−5
0
5
10
15
Surface-to-Surface exact Euclidean Distances (mm)

T2.5

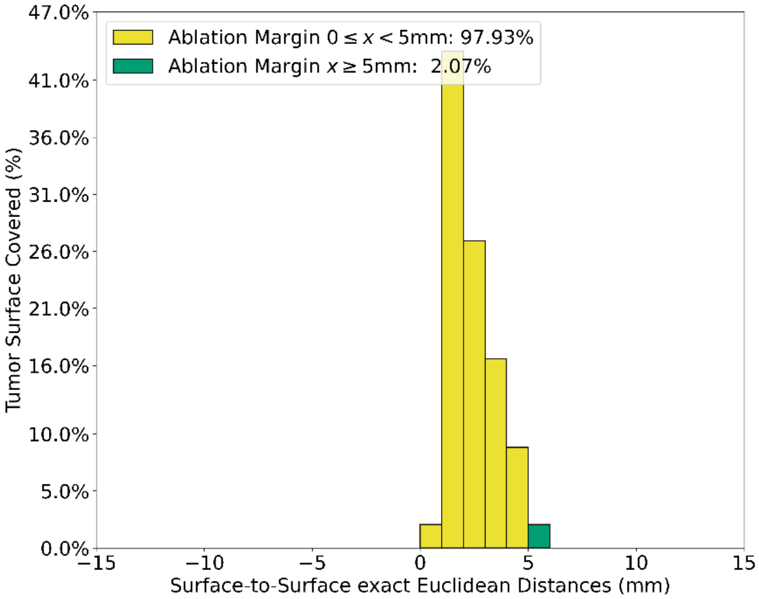

Ablation Margin 0 ≤ x < 5mm: 97.93%
Ablation Margin x ≥ 5mm: 2.07%
47.0%
41.0%
36.0%
31.0%
26.0%
21.0%
16.0%
10.0%
5.0%
0.0%
Tumor Surface Covered (%)
−15
−10
−5
0
5
10
15
Surface-to-Surface exact Euclidean Distances (mm)

T2.6

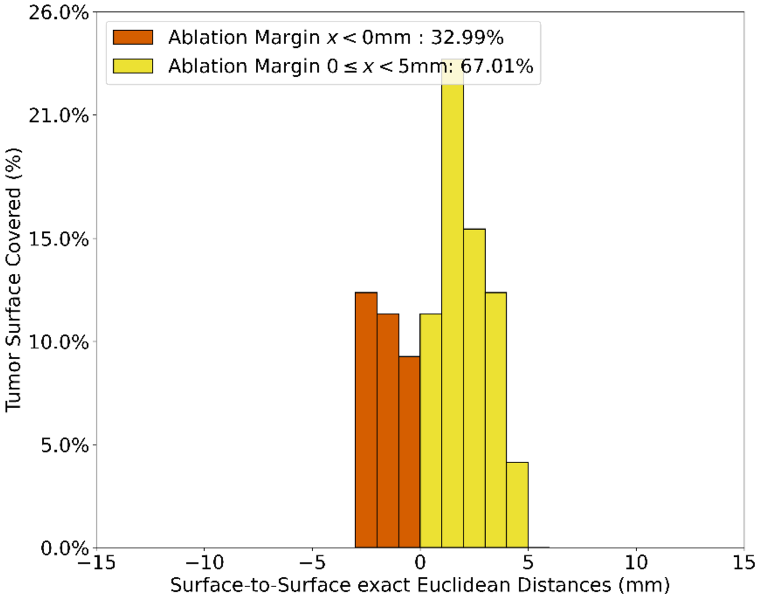

Ablation Margin x < 0mm : 32.99%
Ablation Margin 0 ≤ x < 5mm: 67.01%
26.0%
21.0%
15.0%
10.0%
5.0%
0.0%
Tumor Surface Covered (%)
−15
−10
−5
0
5
10
15
Surface-to-Surface exact Euclidean Distances (mm)

T2.7

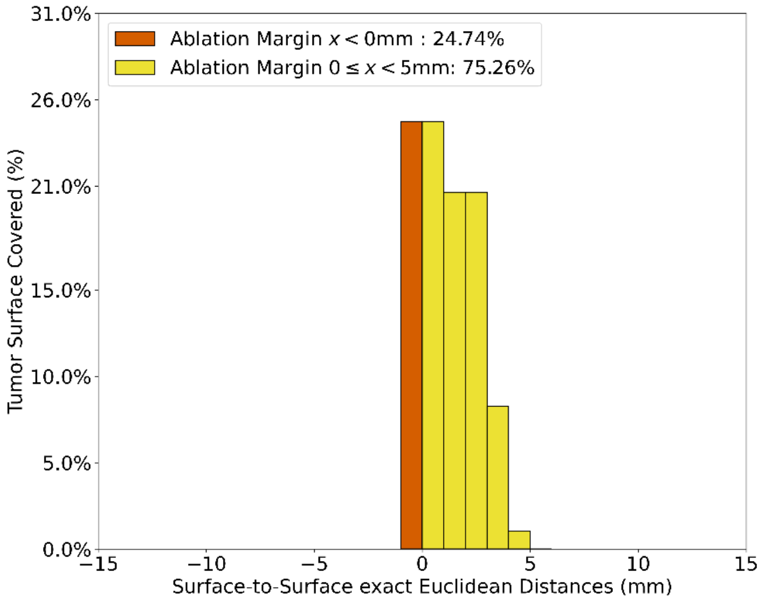
Ablation Margin x < 0mm : 24.74%
Ablation Margin 0 ≤ x < 5mm: 75.26%
Tumor Surface Covered (%)
31.0%
26.0%
21.0%
15.0%
10.0%
5.0%
0.0%
−15
−10
−5
0
5
10
15
Surface-to-Surface exact Euclidean Distances (mm)

T2.8

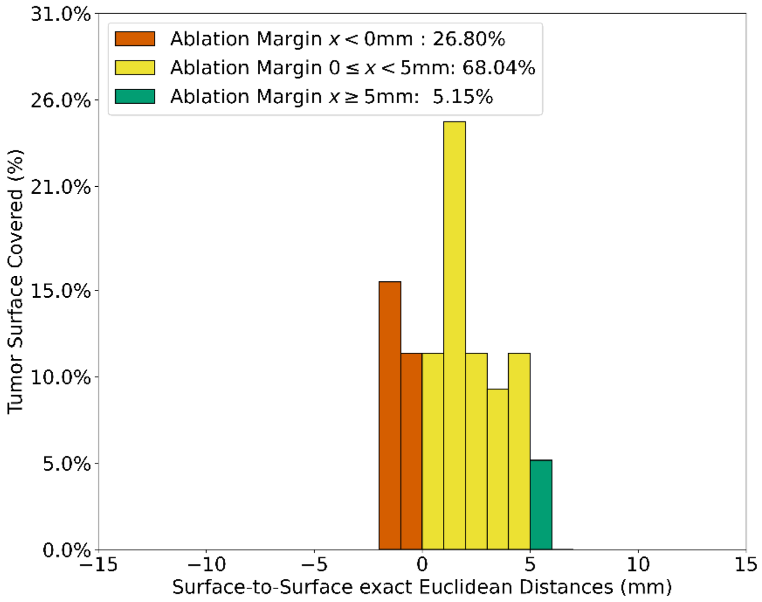
Ablation Margin x < 0mm : 26.80%
Ablation Margin 0 ≤ x < 5mm: 68.04%
Ablation Margin x ≥ 5mm: 5.15%
Tumor Surface Covered (%)
31.0%
26.0%
21.0%
15.0%
10.0%
5.0%
0.0%
−15
−10
−5
0
5
10
15
Surface-to-Surface exact Euclidean Distances (mm)

T2.9

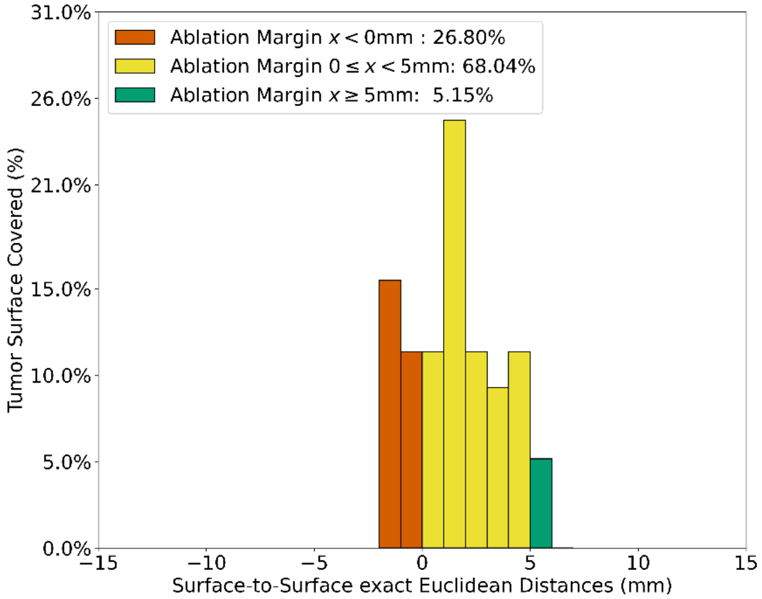

Ablation Margin x < 0mm : 26.80%
Ablation Margin 0 ≤ x < 5mm: 68.04%
Ablation Margin x ≥ 5mm: 5.15%
Tumor Surface Covered (%)
31.0%
26.0%
21.0%
15.0%
10.0%
5.0%
0.0%
−15
−10
−5
0
5
10
15
Surface-to-Surface exact Euclidean Distances (mm)
